# Neglected Anterior Dislocation of the Knee with Common Peroneal Palsy

**DOI:** 10.1155/2015/174965

**Published:** 2015-06-15

**Authors:** Thomas Matthai, Kaushik Bhowmick, P. R. J. V. C. Boopalan, James C. George

**Affiliations:** ^1^Department of Orthopaedics, Christian Medical College, Vellore 632004, India; ^2^Muthoot Medical Centre, Pathanamthitta, Kerala 689641, India

## Abstract

Knee dislocations usually follow high velocity injuries and are increasingly being treated with immediate reduction and staged repair of the ligaments. Neglected knee dislocations are rare and more difficult to treat with inferior outcomes. We present a rare case of neglected anterior dislocation of the knee treated by surgical arthrodesis.

## 1. Introduction

Traumatic dislocation of the knee is both a rare and severe injury. Knee dislocations are classified by the direction of the tibial displacement with respect to the femur as anterior, posterior, medial, lateral, and rotary, with anterior dislocation being the commonest [1]. Knee dislocations are associated with multiple ligamentous injury and neurovascular compromise and hence warrant emergency reduction of dislocated knee [2]. Neglected knee dislocations are not commonly seen. The few reported cases of neglected knee dislocations are posterior [3, 4]. We present hitherto an unreported case of neglected anterior dislocation of the knee with common peroneal palsy.

## 2. Case Report

A 20-year-old manual labourer presented to the outpatient department with complaints of pain and deformity of his right knee and inability to weight-bear on the right leg. He fell into a gutter 1 year back prior to presentation to the hospital. He had taken initial treatment in the form of oil massage and indigenous splinting from traditional bone setters for 45 days.

**Figure 1:**
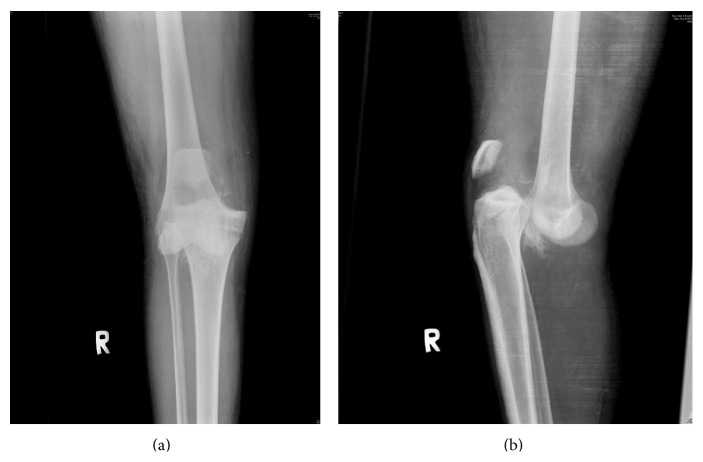
Anterior subluxation of tibia on femur.

Physical examination revealed an unreduced anterior dislocation of the knee with instability in all planes and a painful range of knee movement from ten degrees of hyper extension to ten degrees of flexion. He had associated common peroneal nerve (CPN) palsy. Radiographs confirmed an unreduced anterior dislocation of knee (Figures 1(a) and 1(b)).

The confounding factors in this case were heterotopic ossification between the posterior tibia and femur and common peroneal nerve palsy with possible chondrolysis of the unreduced knee cartilage. After weighing the options, he was planned for an open reduction of the knee with staged ligament reconstruction.

**Figure 2:**
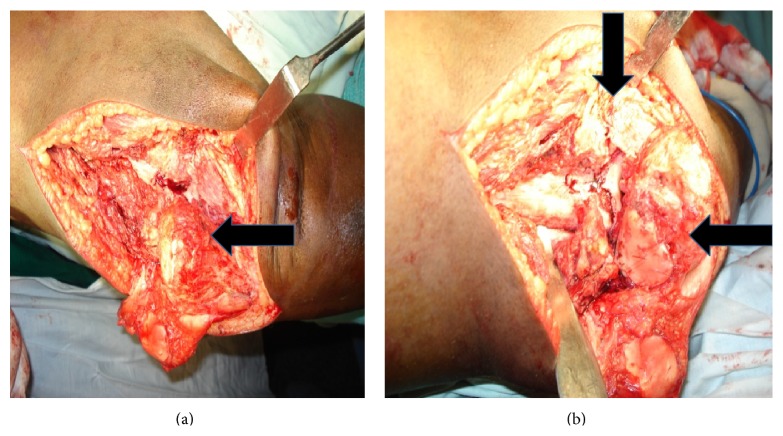
Chondrolysis of femoral and tibial condyles.

Intraoperative exploration involved release of collateral ligaments, popliteus, and the patellar tendon. In spite of extensive release of soft tissues, reduction of the femur onto the tibia was not possible. In addition, there was extensive chondrolysis of the articular surfaces (Figures 2(a) and 2(b)). The cruciate ligaments and the menisci were irretrievably damaged. Hence, an intraoperative decision to arthrodese the knee was made. Resection of overlapping edges of the distal femur and proximal tibia was done for approximation and alignment.

**Figure 3:**
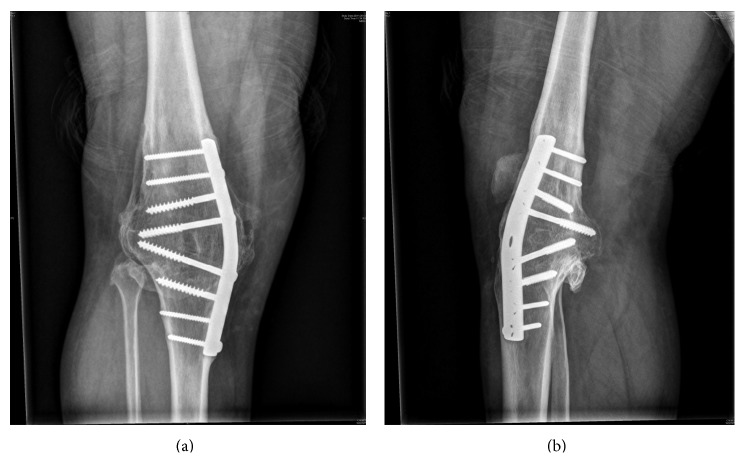
Arthrodesis with contoured DCP.

Primary arthrodesis of right knee with a contoured DCP was done (Figures 3(a) and 3(b)). Exploration of the CPN revealed no discontinuity and neurolysis was done. Postoperatively, the patient was non-weight-bearing with axillary crutches for 6 weeks followed by progressive ambulation. His CPN palsy recovered completely at six weeks following surgery. At 2 years of follow-up, patient had minimal functional disability and was able to pursue his previous occupation as a manual labourer. His modified knee society score was 65 out of 75 (25 points for knee ROM were excluded as his knee was arthrodesed) [5].

## 3. Discussion

Knee dislocations are classically defined as a complete loss of the tibiofemoral articulation that is confirmed radiographically [1]. These injuries have been considered to be the result of high energy trauma and are believed to be rare [6]. While recent literature suggests a staged and early repair of the ligaments produces better results [7] than late repair, there is no much information available on management of neglected dislocation. Moreover, we could not find any previous articles reporting neglected anterior dislocations of the knee in English literature.

In patients with knee dislocations and ruptured common peroneal nerve, even on repair, complete recovery is usually rare. In patients with intact common peroneal nerve, the mode of injury has been described as being due to traction forces, with severity of injury being directly proportional to the injury to the surrounding soft tissue and intra-articular structures. Intraoperatively, the evidence of injury to the nerve is attenuation, oedema, perineural haematoma, and contusion. Even then, complete recovery is seen in 20–30% of these patients. One factor which has been described is the length of the nerve contused, with <7 cm having higher chances of recovery [8, 9].

This patient presented with chronic unstable dislocated knee over one year with CPN injury. Intraoperatively, the nerve was intact and contusion was mild. The recovery after the neurolysis can probably be explained by the reduction in traction due to the multidirectional instability reduced by arthrodesis of the knee.

Mechanism of injury of knee dislocation has been reported to be from violent trauma usually. This case was unusual both in the rarity and the mode of injury, which though trivial (fall in a gutter) probably produced a hyperextension force causing the knee dislocation. Arthroplasty and arthrodesis [10, 11] are the two conventional modes of treatment of unreduced knee dislocations. The former was not considered in this patient because of the possible need for revision in him since he is a manual labourer. Though staged reduction followed by reconstruction of ligaments has been described previously for an unreduced posterior dislocation of knee of up to 6 months of duration, for this patient we considered arthrodesis to be the most appropriate management because of the damaged cartilage of the articular surfaces and the presence of exuberant ectopic bone which hampered reduction. Most importantly, the patient was a manual labourer who needed to get back to work as soon as possible and a painless stable knee joint seemed to be the ideal solution.
